# Neglected Tropical Diseases of the Middle East and North Africa: Review of Their Prevalence, Distribution, and Opportunities for Control

**DOI:** 10.1371/journal.pntd.0001475

**Published:** 2012-02-28

**Authors:** Peter J. Hotez, Lorenzo Savioli, Alan Fenwick

**Affiliations:** 1 Departments of Pediatrics and Molecular Virology & Microbiology, and National School of Tropical Medicine, Baylor College of Medicine, Houston, Texas, United States of America; 2 Sabin Vaccine Institute and Texas Children's Hospital Center for Vaccine Development, Houston, Texas, United States of America; 3 Department of Control of Neglected Tropical Diseases, World Health Organization, Geneva, Switzerland; 4 Schistosomiasis Control Initiative and Department of Infectious Disease Epidemiology, Imperial College, St. Mary's Campus, London, United Kingdom; Yale School of Public Health, United States of America

## Abstract

The neglected tropical diseases (NTDs) are highly endemic but patchily distributed among the 20 countries and almost 400 million people of the Middle East and North Africa (MENA) region, and disproportionately affect an estimated 65 million people living on less than US$2 per day. Egypt has the largest number of people living in poverty of any MENA nation, while Yemen has the highest prevalence of people living in poverty. These two nations stand out for having suffered the highest rates of many NTDs, including the soil-transmitted nematode infections, filarial infections, schistosomiasis, fascioliasis, leprosy, and trachoma, although they should be recognized for recent measures aimed at NTD control. Leishmaniasis, especially cutaneous leishmaniasis, is endemic in Syria, Iran, Iraq, Libya, Morocco, and elsewhere in the region. Both zoonotic (*Leishmania major*) and anthroponotic (*Leishmania tropica*) forms are endemic in MENA in rural arid regions and urban regions, respectively. Other endemic zoonotic NTDs include cystic echinococcosis, fascioliasis, and brucellosis. Dengue is endemic in Saudi Arabia, where Rift Valley fever and Alkhurma hemorrhagic fever have also emerged. Great strides have been made towards elimination of several endemic NTDs, including lymphatic filariasis in Egypt and Yemen; schistosomiasis in Iran, Morocco, and Oman; and trachoma in Morocco, Algeria, Iran, Libya, Oman, Saudi Arabia, Tunisia, and the United Arab Emirates. A particularly noteworthy achievement is the long battle waged against schistosomiasis in Egypt, where prevalence has been brought down by regular praziquantel treatment. Conflict and human and animal migrations are key social determinants in preventing the control or elimination of NTDs in the MENA, while local political will, strengthened international and intersectoral cooperative efforts for surveillance, mass drug administration, and vaccination are essential for elimination.

## Introduction

The neglected tropical diseases (NTDs) are a group of 17 or more chronic parasitic diseases and related infections that represent the most common illnesses of the world's poorest people [1]. An important feature of the NTDs is their ability to promote poverty because of their impact on child development, pregnancy outcome, and worker productivity [2]. Another distinguishing feature is how they vary in their etiologies, prevalence, and disease burden based on their geographic distribution. The prevalence and distribution of the NTDs in the Americas [3]–[5], Europe [6], sub-Saharan Africa [7], China and East Asia [8], India and South Asia [9], and Central Asia [10] have been reviewed previously. Here, we summarize current knowledge on the prevalence and distribution of the NTDs in the Middle East and North Africa (MENA), focusing on aspects particular to the region. The review of the literature was conducted using the online database PubMed from 2003 to 2011 with Medical Subject Headings, the specific diseases listed in the World Health Organization's first report on NTDs [1], and the geographic regions and countries of MENA. Reference lists of identified articles and reviews were also hand searched as were databases from the World Health Organization (WHO, http://www.who.int), including the WHO's Weekly Epidemiological Record.

## Overview of the Middle East and North Africa

**Figure 1:**
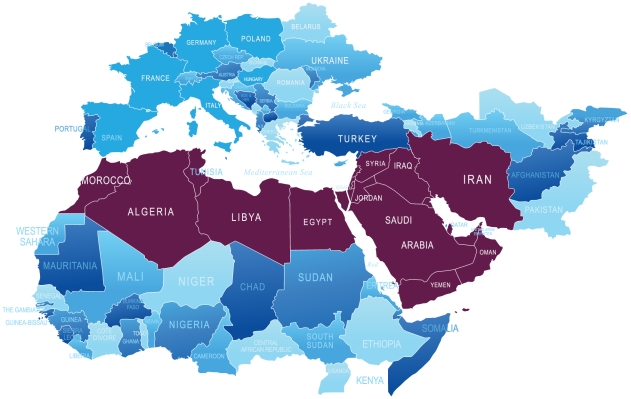
Map of the Middle East and North Africa region.

Approximately 20 countries comprise the MENA as defined by the World Bank [11] (Figure 1). Since the first quarter of 2011, the MENA has undergone sweeping political changes, with major reforms in Tunisia and Egypt and internal strife in Syria, some of the Gulf states, Libya, and Yemen [11]. Almost 400 million people, approximately 5% of the world's population, live in the MENA, led by Egypt (80 million), Iran (75 million), Algeria (36 million), and Morocco and Iraq (31–32 million each) as the most populated countries [12] (Table 1). Estimates from 2005 indicate that 3.6% of the MENA population lives below the World Bank poverty figure of US$1.25 per day, while 16.9% lives below US$2 per day [13]. These “bottom 14 million” and “bottom 65 million”, respectively, are the groups of people with the greatest vulnerability to the NTDs. As shown in Table 1, Yemen has the highest percentage of people living in poverty of all the MENA countries, with Egypt representing the nation with the largest total number of people living in poverty [12]. Significant numbers of impoverished people also live in Algeria, Djibouti, Iran, Iraq, Morocco, and Tunisia [12], [14]. Most of these countries are classified by the World Bank as lower-middle-income countries [15]. In addition to poverty, chronic conflict in the Middle East and associated breakdowns in public health and animal control have contributed to the emergence of NTDs in the region [16].

**Table 1:** Population of the countries of the MENA region and percentage living in poverty.

Country	Total Population	Percentage of the Population Living on Less Than US$2 per Day
Algeria	36.0 million	24%
Bahrain	1.3 million	
Djibouti	0.9 million	
Egypt	80.4 million	18%
Iran	75.1 million	08%
Iraq	31.5 million	06%
Israel	7.6 million	
Jordan	6.5 million	04%
Kuwait	3.1 million	
Lebanon	4.3 million	
Libya	6.6 million	
Malta	0.4 million	
Morocco	31.9 million	14%
Oman	3.1 million	
Palestinian Territory	4.1 million	
Qatar	1.7 million	
Saudi Arabia	29.2 million	
Syria	22.5 million	
Tunisia	10.5 million	13%
United Arab Emirates	5.4 million	
Yemen	23.6 million	47%
TOTAL MENA	392 million	16.9%

Based on reference [12]. Data for poverty in Iraq from [14]. When no number appears it indicates that the data is not available.

Shown in Table 2 is a ranking of the most common NTDs in the MENA, led by ascariasis (a soil-transmitted nematode infection commonly known as roundworm) and schistosomiasis, which are followed by the other soil-transmitted nematode infections (commonly known as hookworm and whipworm), fascioliasis, trachoma, leishmaniasis, and leprosy. Egypt and Yemen—the two countries with the largest number of people living on less than US$2 per day—stand out for ranking first and second, respectively, in having the greatest number of cases of ascariasis, schistosomiasis, fascioliasis, and leprosy (Table 3). They may also represent the last remaining MENA countries with endemic lymphatic filariasis (LF), although each country has recently stopped mass drug administration against LF, having achieved possible elimination. In addition, Egypt has the largest number of hookworm cases (although the prevalence is not high due to the dry conditions) and ranks second in trichuriasis, while Yemen is the only MENA country with endemic onchocerciasis. Algeria, Iran, Libya, Morocco, and Syria follow Egypt and Yemen as impoverished countries with high rates of NTDs. Algeria ranks second in trachoma and fourth in schistosomiasis; Iran, which leads the MENA in cases of zoonotic cutaneous leishmaniasis (CL) (*Leishmania major* infection), is second in CL (*Leishmania tropica* infection) and third in ascariasis, trichuriasis, fascioliasis, and leprosy; Libya is now third or fourth in schistosomiasis and trachoma; Morocco has the highest rates of trichuriasis and is third or fourth in ascariasis, anthroponotic CL, and leprosy, but has eliminated schistosomiasis. Saudi Arabia also has high rates of zoonotic CL, schistosomiasis, and hookworm, in addition to dengue and Rift Valley fever. Leishmaniasis (both CL and visceral leishmaniasis [VL]) and other NTDs are endemic in Iraq, in part because of breakdowns in public health infrastructure and 8 years of war [17]. However, precise estimates of the number of cases are often not available, as is the case for many NTDs in the MENA.

**Table 2:** Ranking of NTDs in the MENA region by prevalence.

Disease	Estimated or Reported Number of Cases	Percentage of Global Burden of Disease	Reference
Ascariasis	22.3 million	3%	[18]
Schistosomiasis	12.7 million	6%	[38]
Trichuriasis	9.0 million	1%	[18]
Hookworm	4.7 million	1%	[18]
Fascioliasis	0.9 million	36%	[43]
Trachoma	0.6 million	1%	[59]
Anthroponotic cutaneous leishmaniasis (*L. tropica*)	0.04 million	Not determined	[48]
Zoonotic cutaneous leishmaniasis (*L. major*)	0.03 million	Not determined	[48]
Leprosy	<0.01 million	3%	[63]
Rift Valley fever	>1,000 cases during outbreaks	Not determined	[68]
Brucellosis	Not determined	Not determined	
Dengue	Not determined	Not determined	
Echinococcosis	Not determined	Not determined	
Crimean Congo hemorrhagic fever	Not determined	Not determined	
Alkhurma hemorrhagic fever	Not determined	Not determined	
Toxoplasmosis	Not determined	Not determined	
Visceral leishmaniasis	Not determined	Not determined	

**Table 3:** MENA countries with the highest prevalence of NTDs.

Disease	Estimated or Reported Number of Cases	Country with Highest Prevalence	Country with Second Highest Prevalence	Country with Third Highest Prevalence	Country with Fourth Highest Prevalence	Reference
Ascariasis	22.3 million	Egypt, 8.3 million	Yemen, 5.8 million	Iran, 5.1 million	Morocco, 1.3. million	[18]
Schistosomiasis	12.7 million	Egypt, 7.2 million	Yemen, 2.9 million	Algeria, 2.3 million	Libya, 0.3 million	[38]
Trichuriasis	9.0 million	Morocco, 3.2 million	Egypt, 1.7 million	Iran, 1.6 million	Yemen, 1.5 million	[18]
Hookworm	4.7 million	Egypt, 3.6 million	Iran, 0.4 million	Saudi Arabia, 0.4 million	Oman, 0.2 million	[18]
Fascioliasis	0.9 million	Egypt, 830,000	Yemen,37,000	Iran, 10,000	Not determined	[43]
Trachoma	0.6 million	Yemen, 204,984	Algeria, 143,356	Iraq, 140,697	Libya, 24,244	[59]
Anthroponotic cutaneous leishmaniasis (*L. tropica*)	0.04 million	Syria, 27,739	Iran, 8,649	Morocco, 1,697	Yemen, 179	[48]
Zoonotic cutaneous leishmaniasis (*L. major*)	0.03 million	Iran, 18,175	Saudi Arabia, 4,238	Tunisia, 2,750	Morocco, 3,431	[48]
Leprosy	<0.01 million	Egypt, 912	Yemen, 424	Iran, 81	Morocco, 72	[63]

## Helminthic NTDs

The most common helminthic NTDs are the soil-transmitted helminthiases, schistosomiasis, fascioliasis, and echinococcosis. LF is undergoing elimination in the region as a result of mass drug administration.

### Soil-Transmitted Nematode Infections

Soil-transmitted nematode infections are the most common NTDs in the MENA, led by 23 million cases of ascariasis, 9 million cases of trichuriasis, and 4–5 million cases of hookworm infection, each representing 1%–3% of the global disease burden from these conditions [18]. More recent estimates are pending as part of the new Global Helminth Atlas [19]. Among the MENA countries, Egypt leads in the number of cases of ascariasis and hookworm infection [18]. Relative to elsewhere in the world, the hookworm situation in Egypt is unusual in that most of the indigenous infections are caused by *Ancylostoma duodenale* rather than *Necator americanus* [20]. Trichuriasis rates are highest in Morocco, followed by Egypt [18]. Enterobiasis, hymenolepiasis, and strongyloidiasis are also found in some surveys, although there are not detailed estimates of the number of cases. There is marked variation in the prevalence of the major soil-transmitted nematode infections, depending on the level of economic development and levels of rainfall and moisture. In Libya, ascariasis, enterobiasis, and hymenolepiasis are associated with lack of education, low socioeconomic status, and family size [21]. In Iran the overall national prevalence of these infections is low, with overall rates declining over the past few years, except possibly for enterobiasis [22]. In Yemen, soil-transmitted nematode infections are common and associated with gastrointestinal symptoms, but they are not necessarily linked to malnutrition [23], [24]. Toxocariasis has been found in Egypt [25]–[27] and Iran [28], and presumably elsewhere in the MENA. Overall, the mass drug administration coverage for soil-transmitted nematode infections is low, with only 4% of both school-aged children and pre-school children at risk in the WHO-designated Eastern Mediterranean Region receiving periodic anthelminthic therapy with benzimidazoles [29].

### Filarial Infections: Lymphatic Filariasis and Onchocerciasis

LF was endemic in two countries in the MENA, Egypt and Yemen, and small foci of infection may remain in Djibouti and Saudi Arabia [30]. Yemen is the only country in the region that is also co-endemic for onchocerciasis [30]. Oman is considered free of LF, while both Egypt and Yemen have completed five rounds of mass drug administration for LF elimination [30]. Egypt was one of the first countries to implement annual treatment to achieve LF elimination and break transmission; and indeed, transmission was recently shown to be interrupted among villages that prior to mass drug administration exhibited some of the highest rates of LF [30]. A financial assessment revealed that the total costs for these efforts averaged US$1 per individual treated, with more than 75% of the costs provided by the Egyptian government [31]. In some areas of Egypt, only two doses were necessary to eliminate LF [32]. Mass drug administration has also been completed in Yemen and surveillance efforts are underway to determine whether LF has been eliminated there [30]. According to the WHO, all LF mass drug administration areas in Egypt and Yemen have achieved a prevalence of microfilaremia of less than 1%, the threshold necessary for discontinuing mass drug administration and for an assessment of whether elimination has been achieved [33]. An unusual form of onchocerciasis known as “aswad” or “sowda”, which is characterized as a severe dermatitis with edema and secondary bacterial infections, occurs in Yemen and possibly in neighboring southern Saudi Arabia [34]. Civil unrest may cause a break in the elimination efforts in endemic areas of the country.

### Platyhelminth Infections: Schistosomiasis, Fascioliasis, and Echinococcosis

While both urinary tract schistosomiasis (*Schistosoma haematobium* infection) and intestinal schistosomiasis (*Schistosoma mansoni* infection) occur in the MENA, overall the region has seen great progress in the elimination of this disease as a public health problem. Through mass drug administration with praziquantel, together with improvements in economic development in the countries of Iran, Morocco, and Oman, disease elimination is either imminent or it has already occurred [22], [35]–[37]. Similarly, Saudi Arabia has had significant reductions in both forms of schistosomiasis, with an overall prevalence of less than 1% (with approximately one-half of the cases in immigrants) through a control strategy consisting of chemotherapy, use of molluscicides, health education, and access to potable water [36].

Currently, the largest number of cases of schistosomiasis occur in Egypt, Yemen, and Algeria. In Egypt, studies from 2006 indicated approximately 7 million cases of schistosomiasis in that country [38]. Over the last 5 years, however, it is believed those numbers have since decreased, with *S. haematobium* infection almost eliminated and *S. mansoni* infections remaining only in what are described as “hot spots” in the Nile Delta–irrigated area in the northern part of that country. The major interventions responsible for this situation include the building of the Aswan High Dam in 1960, which changed irrigation patterns in the Nile Delta, leading to an improved habitat for *Biomphalaria* snails as opposed to *Bulinus* snails and therefore a reduction in the prevalence of *S. haematobium*, as well as to implementation of mass treatments initially with tartar emetic and subsequently with praziquantel [36]. However, the completion of the Aswan High Dam also had a dark side, with significant increases in the prevalence of *S. mansoni* in the Nile Delta and in new irrigation schemes, in addition to soil erosion and decreased soil fertility, increased salinity, and pesticide pollution of the soil [39], [40]. Many of these effects resulted from the holding back of the silt, which previously had renewed the fertility of the soil in the delta after the annual Nile flood. One important Ministry of Health decision and two important interventions have helped stimulate efforts to eliminate schistosomiasis in Egypt. The decision was to allow mass drug administration using praziquantel. Because of bad experiences with treatment against schistosomiasis using tartar emetic drugs in the 1960s, until 1993 treatment could only be given to individuals diagnosed as infected. The first intervention, started in 1988, was a schistosomiasis research project funded by the United States Agency for International Development and implemented through the Egyptian Ministry of Health and Population [40]. This project led to improved prevalence data, control tools, and capacity for biomedical research focusing on vaccine development, epidemiology, molluscicide studies, social anthropology, and improved diagnostics [40]. The second intervention came in 1997 when the long-running National Schistosomiasis Control Project was boosted by funding from the World Bank to provide praziquantel mass drug administration in schools and in villages where the prevalence exceeded 20% [40]. The trigger level was later reduced to 10% and subsequently to 5%. By the time the program closed in 2002, an estimated 10 million school children at risk in rural Egypt had received praziquantel, and all residents of more than 500 villages at high risk of infection were offered treatment. Significant snail infestations were simultaneously reduced through application of niclosamide [40]. As a result, the prevalence of *S. mansoni* infection was estimated to have decreased from approximately 15% in 1993 to 3% in 2002, decreasing to 1.5% in 2006, and the prevalence of *S. haematobium* infection decreased from 7% in 1993 to 2% in 2002 and then to 1% in 2006 [40]. With these public health gains, the incidence of squamous cell carcinoma of the bladder, a consequence of chronic infection with *S. haematobium*, has fallen precipitously in Egypt [40], [41]. One unfortunate consequence of the early phases of schistosomiasis control with parenteral tartar emetic was the widespread use of improperly sterilized needles, which led to schistosomiasis and hepatitis C co-infections, with elevated viral loads and more rapid progression to cirrhosis and hepatocellular carcinoma infections [42].

Fascioliasis is the second most common trematode infection in the MENA, accounting for more than one-third of the world's cases, most of which occur in Egypt, followed by Yemen and Iran [22], [43]. A possible reason for the high prevalence in Egypt is thought to be the habit of farmers of picking vegetables and then leaving them immersed in the canals to keep them fresh while they continue picking. Treatment of fascioliasis has proved to be difficult, although triclabendazole has proven effective.

Cystic echinococcosis (CE) caused by *Echinococcus granulosus* is the most widespread cestode infection, found throughout the MENA except in the southern Arabian Peninsula. The traditional disdain for dogs in many Muslim communities may limit the overall prevalence and distribution of echinococcosis, but in fact no precise numbers are available regarding the actual number of cases. In Iran, CE is highly endemic and responsible for 1% of all admissions to surgical wards [22]. Between 2001 and 2005 more than 2,000 cases were recorded, but in some regions the seroprevalence of CE exceeds 10% [22]. With regards to the mode of transmission in Iran, there are an estimated 700,000 sheep dogs, of which almost 50% are infected with *E. granulosus*; among the modes of transmission are contamination of raw vegetables, ice cream, and carrot juice with *E. granulosus* eggs [22]. The disease is also found among refugees to Iran from Afghanistan [22]. The sheep (G1) and camel strains are the most common genotypes affecting humans [44], [45]. High levels of infection have also been reported in Algeria and Libya [46], [47].

## Protozoan NTDs

Cutaneous and visceral forms of leishmaniasis are the most important protozoan infections in the MENA, although the actual number of cases is not known due to underreporting [48]. In addition, intestinal protozoan infections are likely to be widespread as is toxoplasmosis, but there is a dearth of information available on the prevalence and distribution of these infections.

### Cutaneous Leishmaniasis

Both zoonotic CL, caused predominantly by *L. major*, and anthroponotic CL, caused by *L. tropica*, are widespread in the MENA. The largest number of *L. major* cases occurs in arid areas of Iran, Saudi Arabia, Morocco, Tunisia, Syria, Libya, and Iraq, with most of the cases transmitted by the sandfly *Phlebotomus papatasi* or closely related species [17], [48]–[51]. In addition to humans, *Ph. papatasi* feed on a variety of mammals and birds, especially a type of gerbil known as the fat sand rat living in the salt flats in an area geographically situated between Morocco, Syria, and Saudi Arabia [48]. The Great gerbil (*Rhombomys opimus*) transmits *L. major* in northwestern Iran and northern Afghanistan [48]. During Operation Iraqi Freedom in 2003, US soldiers received intense exposure to sandflies, incurring more than 600 cases of CL in 2003 and 2004, 99% of which were caused by *L. major* [17]. The largest number of *L. tropica* cases occur in Syria, Iran, Morocco, and Yemen [48], [52], in addition to Algeria [53], where they are transmitted by *Phlebotomus sergenti*, especially in urban areas [50]. Beginning in 2000, CL caused by *L. tropica* emerged in northern Israel, where it is believed the rock hyrax (*Procavia capensis*) may represent an animal reservoir [54], [55]. CL caused by a *Leishmania* species closely related to *Leishmania killicki* has also been reported from Algeria [56]. A major approach to the control of *L. major* infection relies on clearing of vegetation around human habitations and introducing zinc phosphide tablets into gerbil burrow entrances, while control of *L. tropica* infection benefits from indoor residual spraying [48].

### Visceral Leishmaniasis

There are two types of VL: anthroponotic VL caused by *Leishmania donovani* and zoonotic VL caused by *Leishmania infantum*. *L. donovani* infection (transmitted by *Phlebotomus orientalis*) occurs in Yemen and Saudi Arabia [48]. Control relies on case management and treatment with antimonial agents or newer drugs, including liposomal amphotericin B. The dog is the major animal reservoir of *L. infantum*, which is transmitted by several species of sandflies in more than one-half of the MENA countries, including Egypt, Iran, Iraq, Jordan, Lebanon, Libya, Morocco, Saudi Arabia, Syria, Tunisia, and Yemen [17], [48], [57].

### Toxoplasmosis

Toxoplasmosis is another important protozoan infection thought to be present throughout the MENA, although there is a dearth of available information about this disease. The seroprevalence in Lebanon exceeds 60% [58].

## Bacterial NTDs

The most important bacterial NTDs in the MENA are trachoma, leprosy, and brucellosis.

### Trachoma

Morocco was the first nation in the modern era to eliminate trachoma as a public health problem through implementation of the SAFE (surgery, antibiotics, facial cleanliness, and environmental control) strategy with support from the International Trachoma Initiative. According to the WHO Global Health Atlas, more than half a million cases of trachoma occur in the MENA region, with the largest number in Yemen (204,000 cases), followed by Algeria and Iraq (roughly 140,000 cases each) [59]. However, trachomatous trichiasis is still a public health problem among some elderly populations in Oman, particularly in women [60], while trachoma occurs in the Sistan-va-Balucehstan province of Iran [61]. Overall, elimination targets are on track in the nations of Algeria, Iran, Libya, Oman, Saudi Arabia, Tunisia, and the United Arab Emirates [62].

### Leprosy

Leprosy is no longer the scourge of these countries that it once was, and leper colonies in Egypt are being closed. The WHO Regional Office for the Eastern Mediterranean, however, still has an estimated 8,495 registered cases of leprosy, representing approximately 15% of the global leprosy burden [63]. Among countries in the MENA region, Egypt had the largest number of cases with 912, followed by 424 in Yemen, 81 cases in Iran, 72 in Morocco, and 34 in Qatar [63].

### Brucellosis

In the MENA region, conflict, associated breakdowns in veterinary public health systems, and unrestricted animal transportation through open borders have promoted the re-emergence of brucellosis [16]. Among cattle and sheep, the highest prevalence of brucellosis occurs in Jordan, while among goats the highest rates of infection are in Iraq and Jordan, and among camels in Egypt, Iran, and Saudi Arabia [16]. Brucellosis is also an important problem in Libya [64], and it is prevalent among the Bedouin community in Oman [35]. Preventive measures require surveillance, animal control, and increased use of the brucellosis vaccine for animals at risk [16].

## Viral NTDs

During the 1990s, dengue outbreaks began in Djibouti and in Jeddah, Saudi Arabia, for the first time in over 50 years. There have since been outbreaks in both Yemen and Saudi Arabia [65]. Dengue is now endemic in the western and southern regions of Saudi Arabia, with peaks of infection appearing in 2006 and 2008 [66]. Of particular concern are the more than 1 million pilgrims passing through Jeddah placed at risk for dengue while on their way to and from Mecca for the Hajj [66]. Another emerging viral NTD is Crimean-Congo hemorrhagic fever (CCHF), a viral zoonotic disease with a high mortality rate in humans, transmitted through the bite of ixodid ticks [67]. In December 2008, a re-emerging outbreak of CCHF occurred in the southern part of Iran, and livestock were identified as the source of infection [67]. The infection also occurs in Oman [35]. In 2000, Rift Valley fever (transmitted by *Aedes* and *Culex* mosquitoes) emerged in the southwestern part of Saudi Arabia and in the adjacent regions of Yemen [68]. This was the first recorded outbreak outside of Africa, affecting an estimated 40,000 animals and resulting in almost 1,000 reported cases and several hundred deaths [68]. Alkhurma hemorrhagic fever, which is caused by a tick-borne flavivirus, has also emerged on the Arabian Peninsula [69].

## Opportunities for NTD Control in the MENA

There is a dearth of information on many NTDs in the MENA and overall there is an urgent need to expand surveillance efforts for most of the NTDs in this region. Tools are available to control or in some cases eliminate the major NTDs in the MENA. For the soil-transmitted nematode infections, coverage with anthelmintic drugs, especially among school-aged children, needs to increase [29]. Mass drug administration programs with albendazole or mebendazole should be expanded in the high-burden countries of Egypt, Yemen, Morocco, and Iran, although because of post-treatment re-infection there is no evidence that the major soil-transmitted nematode infections will be eliminated in the near future. In contrast, several key helminthic NTDs are near elimination in the MENA. Mass drug administration has now been halted for LF in Egypt and Yemen due to an overall prevalence of less than 1% in these countries [30], [33], while as noted above, schistosomiasis has been eliminated in Iran, Morocco, and Oman. Through mass drug administration with praziquantel, great strides have been made in the control of schistosomiasis in Egypt, with the near elimination of *S. haematobium*–induced bladder cancer [40], although re-emergence is a distinct possibility and *S. mansoni* infection remains endemic in the northern part of Egypt. Schistosomiasis remains endemic in Yemen, although a recent initiative by the country has led to significant World Bank funds being allocated to a 6-year control program in Yemen, which will continue provided the political situation there returns to a level that allows drug distribution. Schistosomiasis also remains in Algeria and Saudi Arabia [36], [38]. Therefore, there is a need to maintain high levels of drug coverage with praziquantel in the region. Onchocerciasis is still endemic in Yemen, the only MENA country with both LF and river blindness. Two zoonotic helminth infections, echinococcosis and fascioliasis, remain highly endemic in much of the MENA, and there are opportunities to exert improved control for these helminthiases through animal treatments and, further downstream, animal vaccination to prevent transmission to humans [70].

CL and VL remain widespread in the MENA. Postigo [48] has recently summarized recommendations for control and elimination efforts for these two highly endemic NTDs. They include implementing training strategies for case detection and treatment and clinical and vector program management, a regionalized system for leishmaniasis surveillance and knowledge sharing, multisectoral collaboration, and an international cooperation [48]. An international research agenda for leishmaniasis in the MENA has also been proposed [71]. However, there are concerns that regional conflicts and associated movements in population may complicate such efforts [72]. For toxoplasmosis, implementing newborn screening may represent an appropriate intervention, but there is a need to better understand the disease burden for this condition in the MENA.

Trachoma has either been eliminated or is close to elimination in eight countries in the MENA [62], and there is a need to extend such efforts to Yemen, the highest disease burden country. Leprosy has been eliminated as a public health problem in all of the MENA countries, although hundreds of cases remain in Egypt and Yemen. Brucellosis may represent the most important bacterial NTD even though it is not on the list of 17 NTDs recognized by the WHO. As for leishmaniasis, international cooperation to enhance surveillance and animal control and to prevent unrestricted animal migrations across borders are required, as is expanded use of brucellosis vaccines for animals at risk of infection [16].

Dengue, CCHF, and Rift Valley fever represent the three most important viral NTDs. Dengue and Rift Valley fever currently are focal problems, especially in Saudi Arabia, and the development and administration of vaccines for these vector-borne diseases, especially to prevent transmission around the time of the Hajj [73], would represent an important public health victory. Alkhurma hemorrhagic fever is also a potential emerging threat during the Hajj [69].

Based on results showing the importance of Egypt and Yemen as the two most highly endemic countries for NTDs, special emphasis for targeted interventions might be considered. An international or regional plan emphasizing Egypt and Yemen for NTD control and elimination, especially for soil-transmitted nematode infections, filarial infections, schistosomiasis, fascioliasis, trachoma, and leprosy, would promote overall disease control in the region and globally. Similarly, expanded international and intraregional cooperation is required for leishmaniasis, brucellosis, and dengue control in the MENA.

**Key Learning Points**

- Neglected tropical diseases (NTDs) are spread out among the 20 countries of the Middle East and North Africa (MENA) region, primarily affecting the estimated 65 million people living on less than US$2 per day.
- Egypt and Yemen have the highest rates of many NTDs, including the soil-transmitted nematode infections, filarial infections, schistosomiasis, fascioliasis, leprosy, and trachoma.
- Leishmaniasis, especially cutaneous leishmaniasis, is endemic in Syria, Iran, Libya, and Morocco. Both zoonotic (*Leishmania major*) and anthroponotic (*Leishmania tropica*) forms are endemic in MENA in rural arid regions and urban regions, respectively.
- Great strides have been made towards elimination of several endemic NTDs, including lymphatic filariasis in Egypt; schistosomiasis in Iran, Morocco, and Oman; and trachoma in Morocco, Algeria, Iran, Libya, Oman, Saudi Arabia, Tunisia, and the United Arab Emirates.
- Conflict and human and animal migrations are key social determinants in preventing the control or elimination of NTDs in the MENA.

**Key Papers in the Field**

- Jacobson RL (2011) Leishmaniasis in an era of conflict in the Middle East. Vector Borne Zoonotic Dis 11: 247–258.
- Memish ZA, Charrel RN, Zaki AM, Fagbo SF (2010) Alkhurma haemorrhagic fever–a viral haemorrhagic disease unique to the Arabian Peninsula. Int J Antimicrob Agents 36 Suppl 1: S53–S57.
- Postigo JA (2010) Leishmaniasis in the World Health Organization Eastern Mediterranean Region. Int J Antimicrob Agents 36 Suppl 1: S62–S65.
- Rokni MB (2008) The present status of human helminthic diseases in Iran. Ann Trop Med Parasitol 102: 283–295.
- Salem S, Mitchell RE, El-Alim El-Dorey A, Smith JA, Barocas (2011) Successful control of schistosomiasis and the changing epidemiology of bladder cancer in Egypt. BJU International 107: 206–211.
